# Low-volume Plasmodium blood sample processing protocols for untargeted transcriptomics optimized using Plasmodium knowlesi

**DOI:** 10.1099/mgen.0.001546

**Published:** 2025-11-20

**Authors:** Erin Sauve, Johanna Helena Kattenberg, Pieter Moris, Pieter Guetens, Pieter Monsieurs, Anna Rosanas-Urgell

**Affiliations:** 1 Malariology Unit, Department of Biomedical Sciences, Institute of Tropical Medicine Antwerp, 155 Nationalestraat, 2000 Antwerp, Belgium

**Keywords:** field studies, parasitology, *Plasmodium*, sample processing, transcriptomics, white blood cell depletion

## Abstract

As the importance of transcriptional variation and regulation for *Plasmodium* becomes more apparent, advances for non-*falciparum* species are hindered by our reliance on natural infections to study parasite biology. Untargeted transcriptomic research is also complicated by low-parasite densities and high proportions of human genetic material, highlighting the need for optimized sample processing protocols. In this study, we used a *Plasmodium knowlesi* culture diluted in whole blood as a mock *Plasmodium vivax* natural infection to compare white blood cell (WBC), rRNA and globin depletion methods and RNA-seq library preparation kits to create an optimized protocol for low-volume sample processing. Depletion techniques and library preparation kit selection are both crucial to enrich parasite RNA in low-parasite density and low-volume samples. WBC depletion methods can increase the percentage of mapped *Plasmodium* paired-end (PE) reads from 5.4 to 37.2–54.5%, depending on the method. Additionally, globin RNA and rRNA depletion significantly decreased the human globin RNA and rRNA combined gene counts up to 90%. When comparing the library preparation kits, the transcription profiles of the mRNA kits were highly correlated and enriched for protein-coding genes. The total RNA library prep kit was dominated by *P. knowlesi* rRNA, despite having the highest percentage of mapped *P. knowlesi* PE reads. Parasite sequencing output can be optimized when WBC and globin depletion are used in combination with an mRNA library preparation kit bringing untargeted *Plasmodium* transcriptomics to resource-limited settings which will lead to new insights in parasite biology.

**Impact Statement**

This study evaluates a combination of white blood cell (WBC) depletion methods and RNA-seq library preparation kits with low-volume samples for untargeted *Plasmodium* transcriptomics. We optimized field-adaptable protocols to generate high-quality RNA-seq data from low-volume *Plasmodium* natural infection samples suitable for low-parasite densities and show the importance of library preparation kit selection and globin, rRNA and WBC depletion methods to increase *Plasmodium* read output. These insights are especially important for *Plasmodium* species which cannot be cultured *in vitro,* with an untargeted transcriptomic approach offering a promising solution to address key biological questions that would otherwise remain unanswered.

## Data Summary

All code, count tables and specific options used for the different steps of the analysis can be found at Zenodo (doi: 10.5281/zenodo.15230922). RNA-seq data (FASTQ files) and expression files of the parasite transcriptome are available in the NCBI GEO (accession: GSE294674).

## Introduction

Transcriptomics is an invaluable tool in *Plasmodium* research as it provides the opportunity to answer biological questions that cannot be answered by genomic studies alone. Investigating the transcriptome has advanced our understanding of parasite gene expression, shedding light on key aspects of parasite biology for drug resistance [1,2], sexual conversion [3,4], host interactions and adaptation to the environment [5,6]. This is particularly valuable for non-*falciparum* species such as *Plasmodium vivax*, which cannot be continuously cultured yet significantly contribute to the global malaria burden. To study *P. vivax* biology, researchers must rely on natural infections and face their inherent challenges, including low-parasite densities and a high proportion of human genetic material. These challenges complicate the extraction of sufficient parasite genetic material and highlight the need for simple, standardized protocols to collect, process and preserve *Plasmodium* natural infection blood samples for RNA-seq.

Without applying depletion methods to natural infection samples, a vast majority of sequencing reads are human*,* resulting in a large amount of unusable data and making it expensive to sequence deep enough for *Plasmodium* differential expression analysis. Applying depletion methods to remove contaminating human RNA and enrich for *Plasmodium* RNA reduces the required sequencing depth. Various white blood cell (WBC) depletion methods have been tested across multiple *Plasmodium* species, including Plasmodipur filters, cellulose columns (previously CF11 columns, now discontinued) and non-woven fabric (NWF) filters. Studies comparing cellulose columns with Plasmodipur filters found that cellulose columns offered effective and more cost-efficient human DNA depletion [7,8], with a slightly better recovery rate of the parasite life stages than Plasmodipur filters [9]. NWF filters, initially proposed as a less-expensive alternative, were comparable to Plasmodipur filters [10] and showed better red blood cell (RBC) recovery than CF11 columns [11]. Unfortunately, these studies focused on DNA and used large-volume venous blood samples (greater than 1 ml); thus, further evaluation is necessary to assess the applicability of these methods to low-volume samples for transcriptomic analysis, such as those collected from a finger-prick. Another widely used depletion method utilizes MACS columns, which deplete WBCs and most RBCs while enriching for late-stage hemozoin-containing parasites. However, this method has a significant drawback since young life stages are lost in the first elution, leaving a substantial portion of the parasite population absent from the sequencing results [12,14].

RNA preservation is a critical aspect of sample collection since RNA is inherently unstable and highly susceptible to degradation compared to DNA. Common preservation methods include RNA preservation buffers and cryopreservation. TRIzol (Invitrogen) has long been a standard in malaria research to preserve genetic material, while newer RNA preservation buffers, such as RNAprotect (Qiagen), offer a quick and simple option that can be easily processed using silica column extraction kits, eliminating the need for toxic and meticulous chloroform extraction protocols that are difficult with low volumes. These reagents have reduced the reliance on cryopreservation for field isolates, with the exception of *ex vivo* studies where it can be helpful to transport parasites from the field to better-equipped laboratories.

For species that can be short-term or continuously cultured such as *P. vivax* and *P. falciparum*, respectively, *ex vivo* maturation is one method to generate sufficient *Plasmodium* material for RNA-seq. However, *ex vivo* maturation is labour, resource and training intensive, making it unfeasible for larger studies. Moreover, it is important to consider the changes in parasite life stage composition as the parasites mature and the potential impact of *ex vivo* maturation on the parasite transcriptome [15,17].

The choice to target either mRNA or total RNA in an RNA-seq experiment depends on the specific biological question being addressed. Total RNA library preparations offer the most extensive transcriptome analysis by capturing all RNA types in a sample, including highly abundant rRNAs and non-coding RNAs important to address expression regulation [18]. In contrast, mRNA library preparations focus exclusively on protein-coding transcripts, delivering more in-depth information into the coding regions of genes ideal for differential gene expression analysis. mRNA library preparations implement poly(A) capture to enrich for mRNA and simultaneously deplete abundant human and *Plasmodium* rRNA, as these lack a poly(A) tail. Another method for depleting unwanted RNAs during library preparation involves using efficient depletion kits to remove specific RNAs, such as globin and human rRNA, which can otherwise dominate sequencing reads [19,23]. While these kits are effective and simplify the removal of unwanted RNAs, a significant downside is their high cost per sample. Regardless, the removal of unwanted RNA transcripts is critical to focus on the sequencing of transcripts of interest and to improve the sensitivity for lowly expressed transcripts.

In this study, we optimized methods to generate high-quality RNA-seq transcriptomes from *Plasmodium* natural infections using a *Plasmodium knowlesi* mock infection. Our aim was to optimize a protocol for low volumes using simple, field-adaptable methods by testing four WBC depletion methods and three library preparation kits. This is essential for *Plasmodium* species which cannot be cultured *in vitro*, such as *P. vivax*, and where untargeted transcriptomic approaches offer a promising solution to address key biological questions that would otherwise remain unanswered.

## Methods

### *P. knowlesi* mock natural infection sample, WBC depletion and RNA preservation

*P. knowlesi* A1-H.1 strain parasites were maintained at 3% haematocrit in continuous culture between 0.5 and 10% parasitaemia in O+ blood and incubated at 37°C under atmospheric conditions of 90% N_2_, 5% O_2_ and 5% CO_2_ as previously described [24]. Parasitaemia was determined using thin smear slides stained with 10% Giemsa and by counting the total number of infected RBC (iRBC) or life stage divided by the total number of RBCs and multiplying by 100%. A minimum of 10,000 RBCs were counted per slide.

A *P. knowlesi* iRBC pellet was diluted to 5,000 parasites/µl (~0.1% parasitaemia) in 5 ml whole blood to simulate a natural infection sample. For the non-filter control samples, 3 aliquots of 250 µl of the mock natural infection sample were immediately stored in 5 vols (1.25 ml) of RNAprotect cell reagent buffer (Qiagen, cat no: 76526) to preserve RNA.

All WBC depletion procedures described below were done in triplicate and three aliquots of each procedure were preserved in 5 vols (V) of RNAprotect buffer.

### Buffy coat removal (cent/pip)

To evaluate the effectiveness of removing the buffy coat as a method of WBC depletion, three 250 µl aliquots of the mock natural infection were spun at 600 g for 10 min to separate the plasma from the iRBC pellet. The plasma and buffy coat were removed with a pipette, and a 100 µl aliquot of iRBC pellet was removed and mixed with 500 µl RNAprotect and stored as the centrifuge/pipette sample.

### Plasmodipur (plasmo)

One 1 ml aliquot of mock natural infection sample was diluted with 5 vols (5 ml) of RPMI, and 1.5 ml of the diluted sample (250 µl iRBC pellet) was pushed through each of the three pre-wet Plasmodipur filter (EuroProxima) using a syringe to create the three aliquots. The three filters were subsequently washed with another 4 ml RPMI. The entire eluate from each Plasmodipur filter was centrifuged at 600 ***g*** for 10 min to pellet the cells, and 100 µl of each of the three pellets was stored in 5 V (500 µl) RNAprotect as the plasmo sample.

### Plasmodipur and MACS

In an effort to enrich for the late life stages, one depletion method included processing half of the samples using a MACS LD column (Miltenyi Biotec) and processing the other half with a Plasmodipur filter (EuroProxima). One 1 ml aliquot of mock natural infection sample was diluted with 5 V (5 ml) of RPMI and divided into three aliquots of 1.5 ml (250 µl iRBC pellet). In triplicate, half of each aliquot (0.75 ml) was pushed through a pre-wet Plasmodipur filter and washed as described above. The other half (0.75 ml) was processed through a pre-wet MACS column attached to a magnet. After the sample passed through, the column was washed two times with 1 ml of MACS buffer (Miltenyi Biotec). The column was removed from the magnet and washed twice with 3 ml MACS buffer, and the final eluate of each aliquot was centrifuged at 600 g for 10 min. The Plasmodipur and MACS (MACS) pellets were then combined, and 100 µl was stored in 5 V (500 µl) RNAprotect buffer as the PMACS sample.

### Cellulose

Three cellulose columns were prepared by hand in a 10 ml syringe, by first adding a double layer of lens paper (Whatman lens cleaning tissue, Grade 105) and sealing the paper with two drops of RPMI. Subsequently, cellulose (Sigma-Aldrich C6288) was added on top of the lens paper and compacted in the column until the 1 ml mark using the plunger. The plungers were carefully removed without disturbing the cellulose, and the columns were pre-wetted with 5 ml of RPMI. One 1 ml aliquot of mock natural infection sample was diluted with 5 V (5 ml) of RPMI, and 1.5 ml of the diluted sample (250 µl iRBC pellet) was added to each of the three columns. The columns were washed with 5 ml of RPMI, and the eluate from each was centrifuged at 600 ***g*** for 10 min to pellet the cells. To preserve the RNA, 100 µl of each pellet was stored in 5 V (500 µl) RNAprotect buffer as the cellulose samples.

### RNA-seq library preparation and sequencing

All samples in RNAprotect buffer were stored at −20 °C for 2 months. RNA was extracted from the samples stored in RNAprotect using the RNeasy Mini extraction kit (Qiagen) and on-column DNA digestion as per the manufacturer’s instructions and eluted in 30 µl of RNase-free water. For each WBC depletion method, 20 µl of each of the triplicates was pooled after extraction, and RNA concentrations were quantified using Qubit RNA High Sensitivity kit (Thermo Fisher Scientific). Between 10.2 and 46.9 ng µl^−1^, total RNAs (Table 1) were recovered per WBC depletion method and the RNA was subsequently divided evenly over three different library preparation kits (Qiagen mRNA, Illumina mRNA, Qiagen total RNA) to evaluate the performance of the different kits.

**Table 1.** Mapping statistics by sample to describe the total number of raw paired-end (PE) reads in millions (M) recovered after sequencing, the total number of PE reads after trimming and the percentage (%) of reads trimmed and the primary mapped PE reads and as a percentage of PE reads after trimming Primary mapped reads are further shown as the number and percentage of primary PE reads mapped to the human and *P. knowlesi* reference genomes, respectively. Lastly, the number of *P. knowlesi* genes was detected and the percentage of genes was covered with at least 10 counts.

Library prep kit	FastSelect depletion	WBC depletion	Total nr of raw PE reads (M)	Total nr of PE reads after trimming (M)	PE reads trimmed (%)	Primary mapped PE reads		Human primary mapped PE reads		*P. knowlesi* primary mapped PE reads		Pk gene detected (≥10 counts)	
						(M)	(%)	(M)	(%)	(M)	(%)	Total	(%)
mRNA_illumina	na	No filter	45.745	44.666	2.4	41.077	92.0	40.373	98.3	0.704	1.7	3,910	76.0
mRNA_illumina	na	Cent/pip	71.653	69.329	3.2	63.040	90.9	61.756	98.0	1.285	2.0	4,309	83.7
mRNA_illumina	na	Plasmo	55.559	51.527	7.3	48.839	94.8	47.430	97.1	1.409	2.9	4,338	84.3
mRNA_illumina	na	PMACS	87.296	82.122	5.9	77.851	94.8	72.296	92.9	5.555	7.1	4,948	96.1
mRNA_illumina	na	Cellulose	104.936	101.058	3.7	95.656	94.7	92.867	97.1	2.789	2.9	4,613	89.6
mRNA_qiagen	Globin	No filter	62.707	30.589	51.2	27.246	89.1	25.763	94.6	1.484	5.4	4,112	79.9
mRNA_qiagen	Globin	Cent/pip	64.670	44.899	30.6	40.726	90.7	37.419	91.9	3.308	8.1	4,622	89.8
mRNA_qiagen	Globin	Plasmo	62.899	21.941	65.1	19.115	87.1	12.005	62.8	7.110	37.2	4,832	93.9
mRNA_qiagen	Globin	PMACS	69.625	13.860	80.1	11.666	84.2	5.953	51.0	5.713	49.0	4,847	94.2
mRNA_qiagen	Globin	Cellulose	62.978	20.674	67.2	18.162	87.8	8.264	45.5	9.897	54.5	4,784	92.9
mRNA_qiagen	Globin and human rRNA	No filter	63.367	14.234	77.5	11.720	82.3	11.060	94.4	0.660	5.6	3,593	69.8
tRNA_qiagen	Globin and human rRNA	No filter	78.580	63.354	19.4	57.156	90.2	41.813	73.2	15.343	26.8	4,182	81.3
tRNA_qiagen	Globin and human rRNA	Cent/pip	54.697	41.159	24.8	37.494	91.1	22.947	61.2	14.547	38.8	4,144	80.5
tRNA_qiagen	Globin and human rRNA	Plasmo	71.054	53.537	24.7	48.897	91.3	12.174	24.9	36.722	75.1	4,548	88.4
tRNA_qiagen	Globin and human rRNA	PMACS	67.631	54.873	18.9	51.196	93.3	10.063	19.7	41.133	80.3	4,727	91.8
tRNA_qiagen	Globin and human rRNA	Cellulose	70.868	45.884	35.3	42.320	92.2	8.614	20.4	33.706	79.6	4,520	87.8

na, not applicable.

**Fig. 1.**
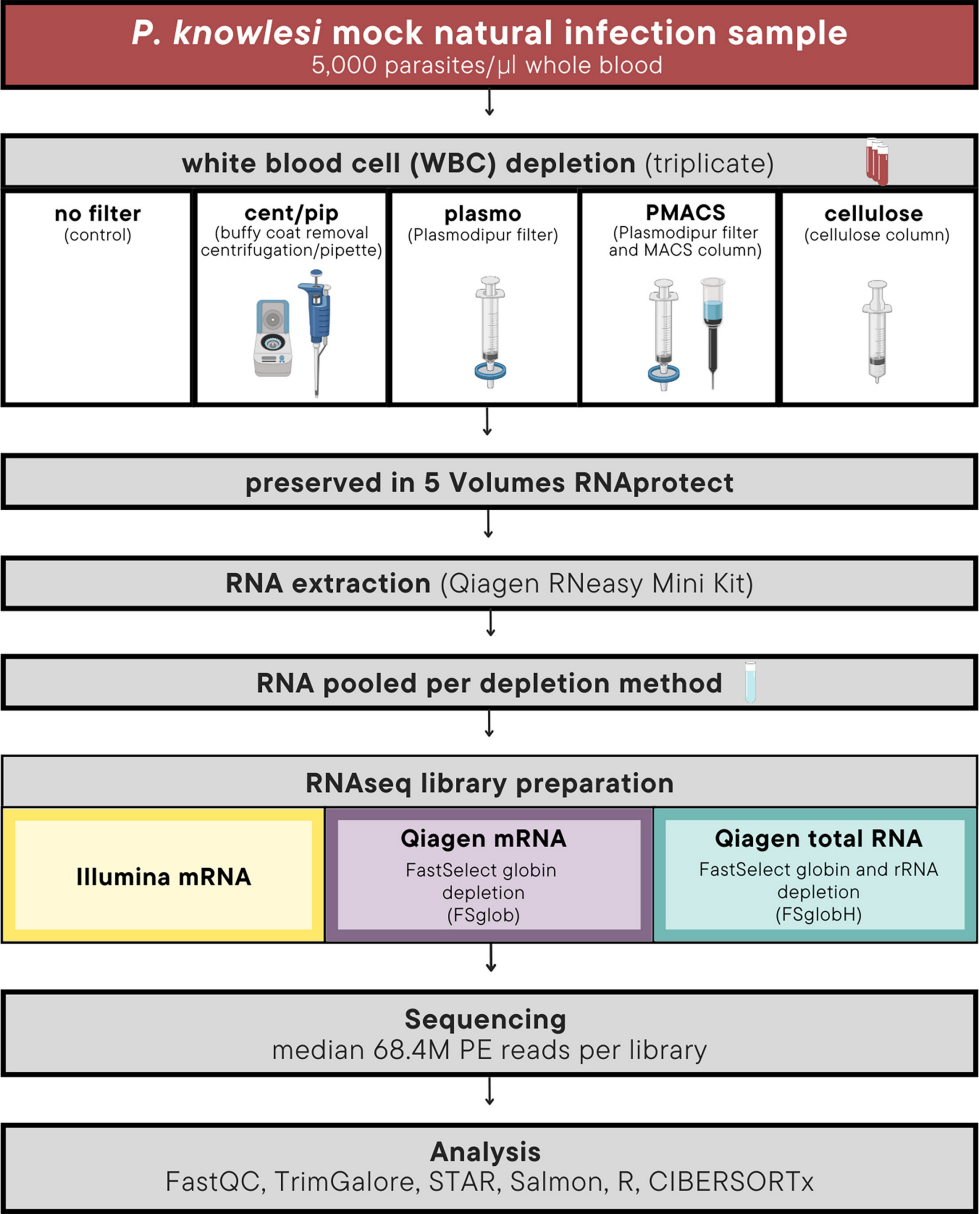
Experimental workflow for testing a *P. knowlesi* mock natural infection using a density of 5,000 parasites per microlitre and four WBC depletion methods [buffy coat removal (cent/pip), Plasmodipur (plasmo), Plasmodipur/MACS column (PMACS) and cellulose column (cellulose)] as compared to a control (no filter). RNA preservation used 5 vols of RNAprotect buffer (Qiagen). RNA extraction was done using the Qiagen RNeasy Mini Kit and library preparation using three kits (Illumina mRNA, Qiagen mRNA and Qiagen total RNA) combined with Qiagen FastSelect globin (FSglob) and FastSelect globin and human rRNA (FSglobH) depletion kits as recommended by the manufacturers. Prepared libraries were sequenced on a NovaSeq 6000 sequencer to generate a median depth of 68.4 million paired-end (PE) reads per library. Analysis was completed using FastQC, TrimGalore, STAR, Salmon, R and CIBERSORTx. WBC depletion method images were created using BioRender.com.

We used the QIAseq Stranded mRNA Lib Kit UDI [24] (Qiagen) for mRNA enrichment of total RNA, fragmentation, reverse transcription, second-strand synthesis+end-repair+A-addition, adapter ligation, CleanStart PCR enrichment and QIAseq Beads for library clean-ups as per the manufacturer’s instructions. For the total RNA library preparation, we used the QIAseq Stranded RNA Lib Kit UDI [24] (Qiagen) as per the manufacturer’s instructions. In addition, in the library preparation steps for these Qiagen libraries, we included the QIAseq FastSelect rRNA HMR and QIAseq FastSelect rRNA globin (Qiagen) for cytoplasmic and mitochondrial ribosomal RNA and globin mRNA removal as per the manufacturer’s instructions. In the total RNA library preparations, we applied both rRNA HMR and rRNA globin FastSelect steps. For the mRNA preparations, we included the rRNA globin FastSelect steps, with the exception of one no filter sample where we applied both FastSelect kits (Fig. 1).

For the Illumina mRNA library preparation, we used the Illumina® Stranded mRNA Prep, Ligation (16 samples) (Illumina), and no globin nor rRNA depletion was included in these library preparations as per the manufacturer’s instructions.

Prepared libraries were quantified using a TapeStation D1000 ScreenTape kit (Agilent) and subsequently sent to a commercial facility for sequencing. Libraries were sequenced on a NovaSeq 6000 sequencer to generate a median depth of 68.4 million paired-end (PE) reads per library (range 45.7–104.9M).

### Analysis of RNA-seq data

The raw reads were screened with FastQ Screen v0.15.3 (in bwa mode) against the human (GRCh38.p14 – RefSeq assembly GCF_000001405.40) and *P. knowlesi* (strain H – RefSeq assembly GCF_000006355.2) reference genomes to assess the proportion of reads derived from each species [25]. The nf-core/rnaseq v3.14 [26] Nextflow workflow [27] of the nf-core collection [28] was then used to process and analyse the reads. Briefly, raw reads underwent FastQC (v0.12.1) quality control [29] and were filtered and trimmed by Trim Galore v0.6.7 [30] and cutadapt v3.4 [31]. Reads were then competitively aligned to the concatenated human and *P. knowlesi* reference genomes using STAR v2.7.9a [32], and gene expression was quantified with salmon v1.10.1 [33] in alignment-based mode. A concatenated version of the human (RefSeq Annotation Release GCF_000001405.40-RS_2023_10) and *P. knowlesi* (GCF_000006355.2 - Apr 3, 2023) annotations was used. Alignment metrics were extracted through samtools v1.20 [34]. Afterwards, the transcriptomic profiles of the different experimental approaches were compared in R v4.4.1 [35]. Reads and transcripts generated by the different methods were compared using chi-square tests. To compare the number of *P. knowlesi* genes with gene counts detected with the different depletion methods, pairwise t-tests with pooled sd and Benjamini–Hochberg (BH) adjustment for multiple testing were used.

Unless specified otherwise, all read count and transcripts per million (TPM) abundance estimates reported are, respectively, gene-level raw counts and abundances obtained by summarizing transcript-level estimates with tximport [36]. Lowly expressed genes were filtered out by only retaining genes for which there were a minimum of ten counts for at least a single sample (reducing the total number of genes down from 55,743 to 27,955, or from 5,452 to 5,147 for *P. knowlesi* genes). Protein-coding and rRNA genes were labelled based on their RefSeq annotation. For human globin, the following genes were selected: ‘HBB’, ‘HBD’, ‘HBBP1’, ‘HBG1’, ‘HBG2’, ‘HBE1’, ‘HBZ’, ‘HBM’, ‘HBA2’, ‘HBA1’ and ‘HBQ1’. For the various visualization of gene counts [heatmaps and principal component analysis (PCA) plots], the filtered raw gene counts were rlog-transformed after subsetting the gene list when appropriate (e.g. species-specific or protein-coding only visualizations).

Raw read counts and TPM values were loaded and visualized in R (v4.4.2) [35] and Inkscape (v1.3.2). Bar plots were created using the packages ggplot2 [37] and viridis [38], and summary values were calculated. Between-sample correlation plots of *P. knowlesi* transcript-level estimates with tximport were visualized using the ggpairs function from the R-package ggplot2 and GGally [39]. PCA and heatmaps were generated using DESeq2 [40] and pheatmap [41].

CIBERSORTx, an analytical tool to deconvolute bulk RNA-seq data to calculate the abundances of cell types in a mixed cell population using a reference matrix, was used to determine the life stage composition of each of our sample RNA-seq data [42]. This was done by inputting our *P. knowlesi* gene-level raw counts into CIBERSORTx along with the *Plasmodium* signature matrix to estimate life stage composition as previously described [43]. Here, we used the b-mode batch correction with 500 iterations as the s-mode batch correction overestimated the gametocyte content of our samples, which we knew was not present due to the nature of *P. knowlesi* A1-H.1 strain [24].

## Results

To optimize *Plasmodium* transcriptome sequencing from low-volume natural infection samples, we tested four WBC depletion methods and three RNA-seq library preparation kits on mock natural infection samples to evaluate WBC, globin RNA and rRNA depletion, as well as *Plasmodium* transcript capture efficiency. We present multiple options for *Plasmodium* RNA-seq that are suitable for field settings, maximize the number of *Plasmodium* reads and minimize unwanted human reads.

### WBC depletion and library preparation approaches impact parasite-specific read output

We evaluated the performance of four WBC depletion methods designed to remove the abundant human genetic material present in whole blood and compared them to a non-WBC-depleted control sample. A mock natural infection sample was prepared by diluting cultured *P. knowlesi* A1-H.1 parasites with human whole blood at a density of 5,000 parasites/µl (~0.1% parasitaemia). The four WBC depletion methods included (1) removing the buffy coat with a pipette after centrifugation (cent/pip), (2) processing the sample with a Plasmodipur filter (plasmo) designed to remove WBCs from malaria-infected blood, (3) combining a Plasmodipur filter with a MACS column (PMACS) to additionally enrich for late life stages and (4) using a homemade cellulose column (cellulose) to remove WBCs (Fig. 1).

RNA was extracted from the samples with recovery between 10.2 and 46.9 ng µl^−1^ total RNA when three replicates from the same depletion procedure were pooled (Table S1, available in the online Supplementary Material). The performance of two library preparation kits targeting mRNA (Illumina and Qiagen) versus total RNA (Qiagen) for the generation of parasite transcriptomes from the WBC-depleted samples was compared (Fig. 1). To eliminate highly abundant contaminating RNA, we tested the impact of a globin and rRNA depletion technology (FastSelect by Qiagen). Removal of rRNA is critical for efficiently targeting desired RNA species in total RNA libraries, as rRNA is highly abundant but of little interest for most research questions. In addition, human globin mRNA transcripts are an important source of contamination in both mRNA and total RNA libraries from human blood samples.

Sequencing libraries were generated with RNA input ranging from 159.6 to 469 ng. The three different library preparation kits resulted in output library concentrations ranging between 0.432 to 10.9 ng µl^−1^ (Table S1). The 16 prepared libraries (5 depletion methods with 3 library preparation kits, plus 1 Qiagen mRNA sample where both the globin and rRNA FastSelect depletion kits were applied) were sequenced to a median depth of 66.2 M PE reads (range 45.7–104.9 M) for each library (Table 1). The sequencing yielded considerably different outputs in terms of good-quality and mapped PE reads depending on the library kit used, despite the libraries being pooled to the same molarity prior to sequencing. The Illumina mRNA kit resulted in the highest total number of PE reads per library after trimming (median 69.3 M PE reads vs 21.3 and 53.5 M PE reads for the Qiagen mRNA and total RNA kits, respectively, Table 1). For the Qiagen kits, a large portion of the reads generated were discarded after trimming [median 35.3% (range 18.9–80.1%) vs the Illumina kit median 3.7% (range 2.4–7.3%), Table 1], due to higher adapter dimer content. When looking at primary mapped parasite-specific PE reads, the Qiagen total RNA kit recovered the most of all three kits tested [median 33.7 M PE reads (range 14.5–41.1 M)]. While comparing the two mRNA kits, the Qiagen mRNA kit yielded more primary mapped parasite-specific PE reads than the Illumina mRNA kit (median 4.5M reads (range 0.66–9.9 M) vs 1.4 M reads (0.70–5.5 M)], despite producing fewer total primary mapped PE reads (Table 1).

Additionally, the WBC depletion methods (plasmo, PMACS and cellulose) significantly enriched for *Plasmodium*-specific reads with a median 32.7% (range 2.9–80.3%) parasite-specific PE reads compared to 5.6% (range 1.7–38.8%) median mapped *P. knowlesi* read pairs for the simplest sample processing approaches (no filter and cent/pip, *χ*^2^ test *P*<0.05; Table 1). The most efficient parasite enrichment was seen in the Qiagen mRNA library preparation kit where the WBC depletion methods led to parasite enrichment from 5.4% with the no filter sample to 54.5%, 49.0% and 37.2% for the cellulose, PMACS and plasmo methods, respectively. Using the Qiagen total RNA library preparation kit, WBC depletion led to the highest overall parasite-specific PE reads (between 75.1 and 80.3%). For the Illumina mRNA kit, only the PMACS depletion method generated considerably more *P. knowlesi*-specific reads than other depletion methods (Table 1).

All sample and library preparation methods detected a high proportion of *P. knowlesi* gene transcripts in the reference genome [median 88.1% of annotated genes with at least ten counts (range 69.8–96.1%)]. All depletion methods (PMACS, cellulose and plasmo) detected more genes than no filter (*P*<0.05, pairwise t-tests with pooled sd and BH adjustment for multiple testing).

### Life stage recovery is consistent across WBC depletion methods except for the PMACS method which enriches for late life stages

**Fig. 2.**
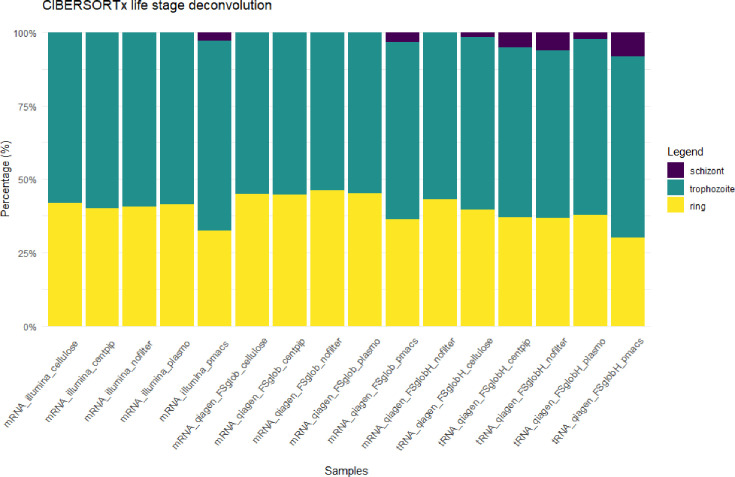
Life stage deconvolution. The life stages recovered including ring (yellow), trophozoite (teal) and schizont (purple) stages by each WBC depletion method and library preparation kit as predicted by bioinformatic deconvolution of RNA-seq data using CIBERSORTx.

The impact of the WBC depletion methods on the recovery of life stages, i.e. life stage composition, was evaluated by microscopy after WBC depletion and *in silico* from the RNA-seq data using CIBERSORTx [42] to deconvolute the parasite transcriptome by life stage [43]. The majority of transcriptomes in all libraries were characterized as trophozoites and rings by both methods. The proportion of rings (range 30.2–46.2%) and trophozoites (range 53.8–64.7%) was stable across most different depletion methods and library kits by CIBERSORTx (Fig. 2). However, these percentages varied by microscopy for rings (range 20–55%) and trophozoites (33–73%) (Table 2). This variability is likely due to differences in transcriptional activity between life stages and species, as previously reported [43,44] and the parasite density of the mock natural infection, which resulted in a low total number of parasites counted per method (range 11–34 parasites), despite counting at least 10,000 RBCs per slide. The life stage compositions determined using CIBERSORTx are also based on the signature matrix which uses the transcriptomic profile of *Plasmodium berghei* and is inherently different from *P. knowlesi*, introducing some variability. Furthermore, we could not investigate the efficiency for gametocyte recovery, as the *P. knowlesi* A1-H.1 strain used does not produce sexual stages [24].

**Table 2.** Microscopy counts before and after WBC depletion per method At least ten thousand RBCs were counted per slide with one slide counted before filtering and three slides being counted per depletion method as the WBC depletion was done in triplicate. Parasitaemia (par %) was calculated by dividing the number of iRBCs by the total number of RBCs. The number of each life stage [ring, trophozoite (troph) and schizont] was counted and the percentage of parasitaemia per life stage (ring %, troph % and schizont %) was determined by dividing the number of each life stage by the number of iRBCs.

Microscopy sample	RBC	iRBC	Par %	Ring	Ring %	Troph	Troph %	Schizont	Schizont %
Before filtering	10,476	11	0.105%	6	55%	5	45%	0	0%
Cent/pip	35,544	18	0.051%	5	28%	10	56%	3	17%
Plasmo	33,108	27	0.082%	16	59%	9	33%	2	7%
PMACS	37,896	34	0.090%	7	21%	19	56%	8	24%
Cellulose	39,300	15	0.038%	3	20%	11	73%	1	7%

Both microscopy and CIBERSORTx results indicate that the PMACS depletion method enriched for the highest percentage of schizonts, with microscopy estimating 24% and CIBERSORTx reporting between 2% and 8% (Table 2, Fig. 2). These results confirm that the magnetic MACS column in the PMACS method takes advantage of the paramagnetic property of the higher hemozoin content in the late life stages [45]. This enrichment is in stark contrast to other methods, such as cellulose columns and Plasmodipur filters, which have been reported to retain late-stage parasites [9,10, 46]. Therefore, if there is particular interest in the late life stages from the original sample, the PMACS method is optimal for enriching for this stage. For studies mainly interested in the overall life stage composition of a sample, or particularly the ring and trophozoite stages, Plasmodipur filters and cellulose columns efficiently deplete WBCs and are time and cost efficient in comparison to the PMACS method. Additionally, it appears that the total RNA kit enriches slightly for the later life stages across depletion methods (Fig. 2), although it is unclear if this is due to an actual enrichment or a compositional bias in the detected reads due to the total RNA kit as the signature matrix used *P. berghei* mRNA single-cell RNA-seq data [43] and therefore may not be as suited for analysing total RNA data.

### Efficient globin and rRNA depletion with FastSelect

To investigate which combination of WBC depletion and library preparation was most efficient in sequencing the parasite transcriptome, the mapped human and *P. knowlesi* gene-level counts of the 16 prepared libraries were compared. Despite implementing WBC depletion methods to remove the majority of human WBC RNA transcripts, high levels of globin RNAs remain present in RBCs and can take up a large proportion of the sequencing output and negatively impact the detection of non-human, non-globin RNAs. To remove unwanted abundant human globin RNA, a FastSelect globin depletion step was added during the Qiagen mRNA and total RNA library preparations. The globin depletion step was not applied to the Illumina library preparations as it was not recommended by the manufacturer.

**Fig. 3.**
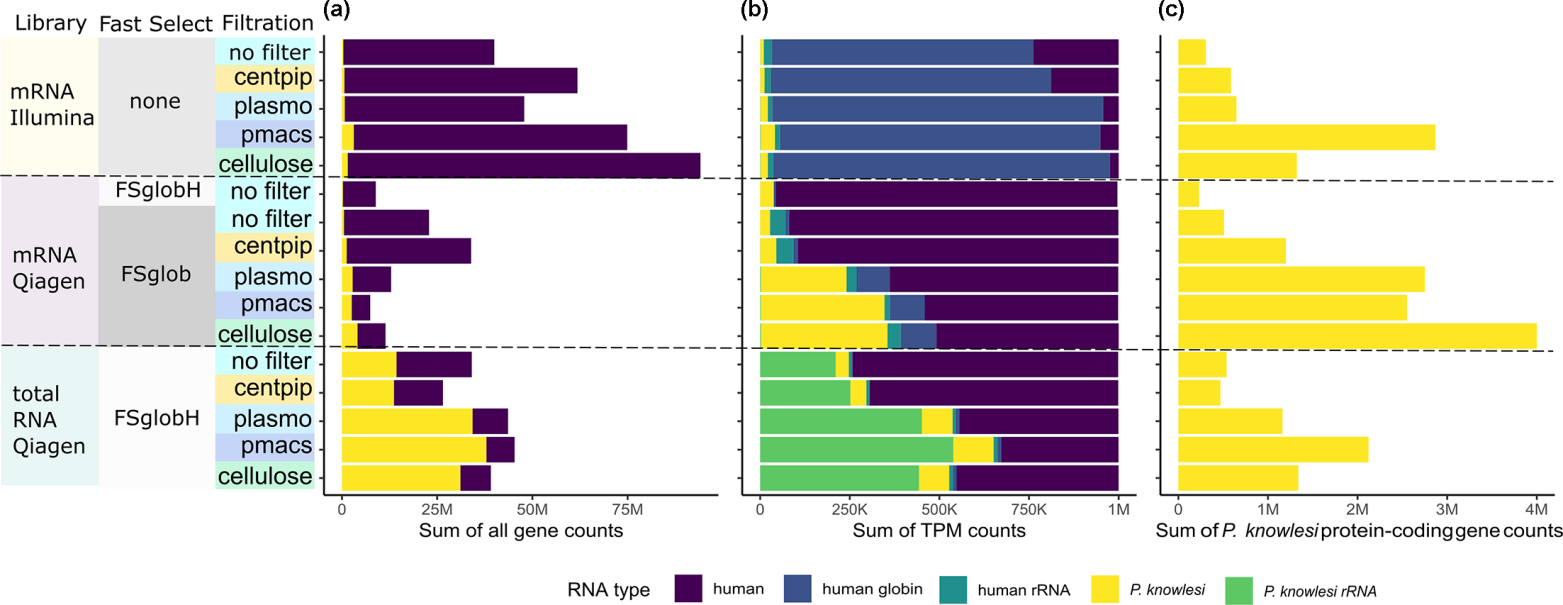
Gene counts per sample. (a) Total number of gene counts per library when mapped competitively to human and *P. knowlesi* reference genomes using Salmon. (**b**) Normalized human and *P. knowlesi* gene TPM counts. (**c**) Total number of *P. knowlesi* gene counts excluding rRNA.

The FastSelect globin depletion included in the Qiagen library preparation kits efficiently removed human globin transcripts, which remained highly abundant in the Illumina mRNA libraries (Table 1, Fig. 3a). While the Illumina mRNA kit generated the highest total number of reads, over 70% of those transcripts derived from the human globin gene (range 729–939 K gene-level TPM counts, Fig. 3b). In contrast, for all libraries where the globin depletion was applied, less than 10% of gene counts were human globin (range 2.5–99 K gene-level TPM counts, Fig. 3b). The Qiagen mRNA libraries had significantly higher proportion of *P. knowlesi* TPM counts than the Illumina mRNA libraries (Qiagen mRNA 27–355 K vs Illumina 10–41 K gene-level TPM counts, *χ*^2^ test *P*<2.2e-16). Globin depletion is thus an essential part of the library preparation of *Plasmodium*-infected blood samples and leads to the highest *P. knowlesi* read recovery (as quantified by gene-level TPM counts), especially when combined with the Plasmodipur, MACS and cellulose column WBC depletion methods (Fig. 3b, c). Despite the high adapter dimer content in the Qiagen mRNA libraries noted above, these libraries had higher *P. knowlesi* protein-coding gene counts than Illumina mRNA libraries, except for the combination of the PMACS depletion in the Illumina library preparation that yielded similar levels of protein-coding gene counts as the Qiagen mRNA library preparation (Fig. 3c).

rRNA is also an abundant contaminating RNA, and thus, its removal is necessary to ensure that predominantly transcripts of interest are sequenced. The FastSelect rRNA depletion step was only added during the total RNA library preparations, as the poly(A) tail capture of the mRNA kit should sufficiently deplete abundant rRNA of any species, with the exception of one Qiagen mRNA control sample (no filter) where rRNA depletion was also applied. This combination resulted in a complete rRNA depletion, while a small proportion of rRNA gene counts remained (<50 K gene-level TPM counts) when this step was omitted from the Qiagen mRNA library preparation (Fig. 3b). The FastSelect rRNA depletion step was not applied to the Illumina mRNA library preparations. For the total RNA libraries, rRNA depletion using FastSelect was more efficient than the poly(A) tail capture in the mRNA kit, as demonstrated by only <10 K gene-level TPM counts of human rRNA remaining in the total RNA library prep kit (Fig. 3b). However, the FastSelect step did not deplete *Plasmodium* rRNA. While the total RNA library preparations did generate a high proportion of *P. knowlesi* transcripts (26.8–80.3%), these were mostly rRNA transcripts (200–500 K gene-level TPM counts) and not protein-coding RNA transcripts (Fig. 3b). For this reason, the total RNA libraries had fewer *P. knowlesi* protein-coding gene counts as compared to the Qiagen mRNA libraries (Fig. 3c).

### Library preparation kit impacts the transcriptome profile more than WBC depletion methods

To investigate which combination of WBC depletion and library preparation leads to the best-quality transcriptomes for expression analyses, gene expression was compared between all tested combinations. As the same mock sample was used in each WBC depletion method and subsequent library preparations, we would ideally expect them to detect similar gene expression patterns after removing contaminating human and parasite RNA types either in the laboratory or *in silico*.

**Fig. 4.**
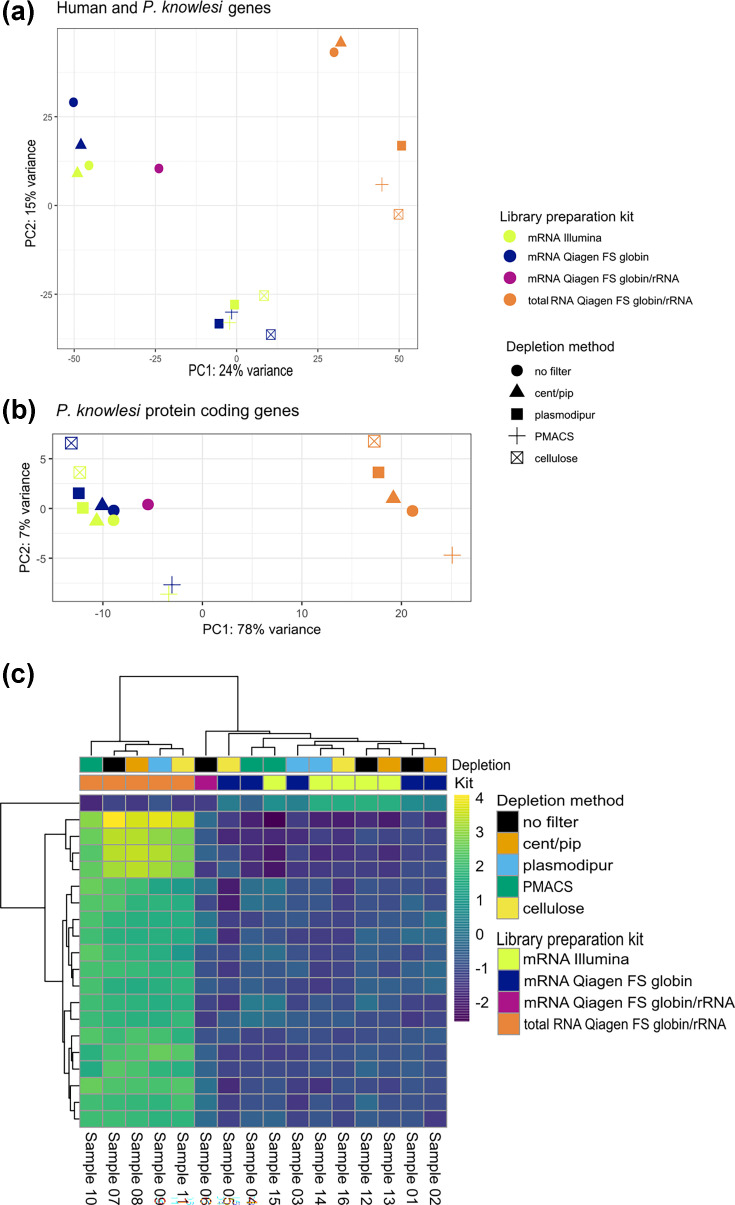
PCA and heatmap plots to visualize the transcriptomes of the different WBC depletion and library preparation kits. (a) Top 500 most variable human and *P. knowlesi* genes. (**b**) Top 500 *P*. *knowlesi* protein-coding genes. (c) Top 20 most variable *P. knowlesi* protein-coding genes. All gene counts were rlog-transformed after subsetting on the gene list of interest. Low counts were filtered prior to subsetting (minimum count of 10 for at least one sample).

First, the transcriptomic profiles of the samples between the three library preparation kits and four WBC depletion methods were visually compared in component analysis (PCA, Fig. 4a, b). When comparing all human and *P. knowlesi* genes, the PCA shows sample separation by kit type and WBC depletion method in the first two dimensions (24 and 15% of variance for PC1 and PC2, respectively), as expected due to their differences in the presence of non-coding RNA and abundance of human reads.

After removing all human and *P. knowlesi* non-coding RNA to look solely at the *P. knowlesi* protein-coding genes, the transcriptomes recovered from both mRNA kits still cluster more closely together and separate from the transcriptomes obtained with the Qiagen total RNA kit (78% of variance explained by PC1 in Fig. 4b). This highlights fundamental differences between total and mRNA library prep kits. The differences can also be clearly observed in the heatmap of the top 20 most variable genes (Fig. 4c) and are further supported by the PCA and heatmap including all *P. knowlesi* RNA (protein-coding and non-coding) which shows high enrichment for non-coding RNAs in the top 20 most variable genes (Fig. S1 and Table S2). In PC2 of Fig. 4b, WBC depletion approaches account for ~7% of the variance, with the biggest difference between transcriptomes of PMACS and cellulose preparations, irrespective of the library preparation approach.

**Fig. 5.**
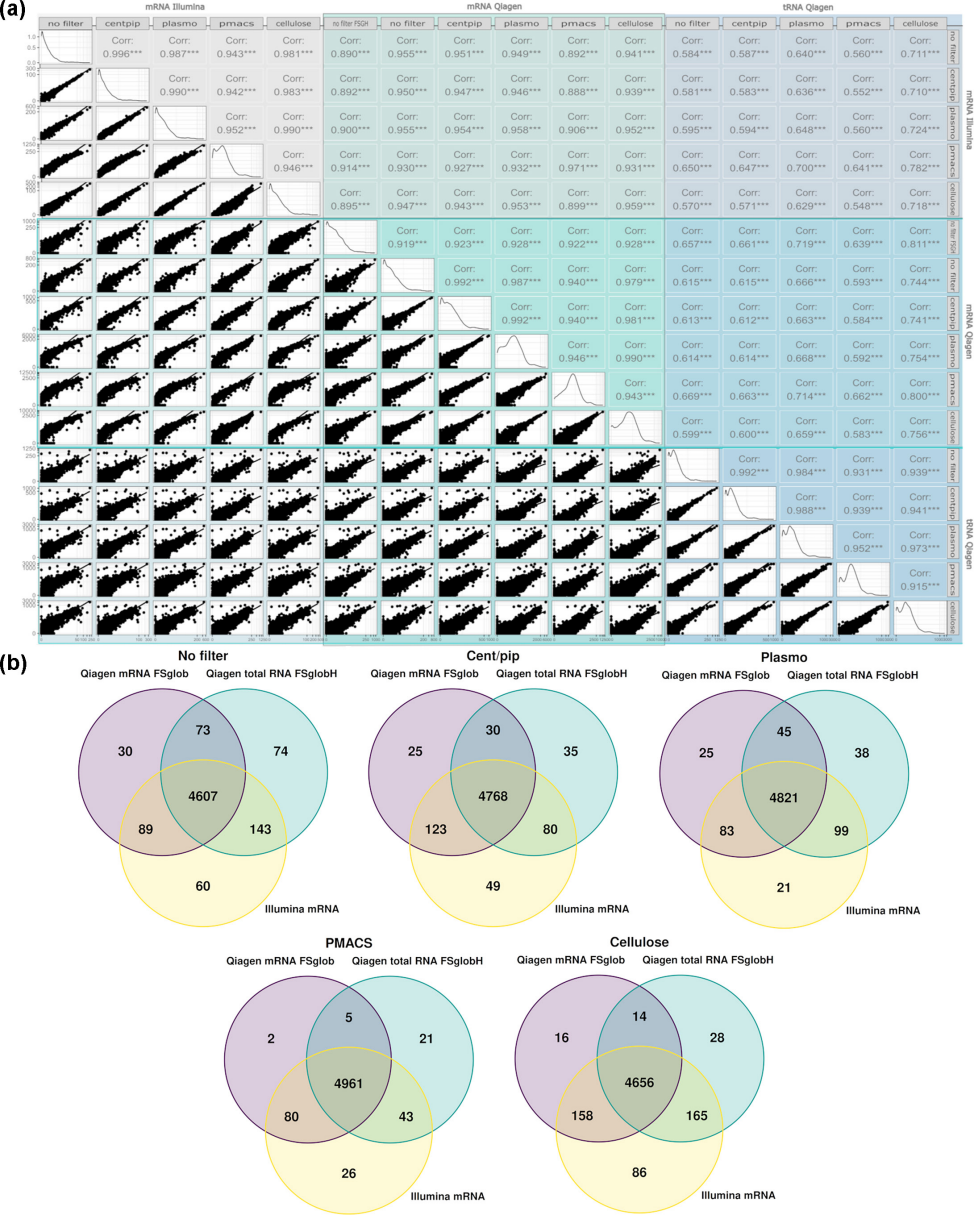
Correlation plots and Venn diagrams to visualize similarities between WBC depletion methods and library preparation kits. (**a**) Correlation plot of TPM abundance estimates of reads mapping to *P. knowlesi* protein-coding genes for each WBC depletion and library preparation method. (**b**) Venn diagrams of *P. knowlesi* genes with >10 counts detected per WBC depletion and library preparation method.

Individual gene expression levels correlated excellently between different WBC depletions when processed with the same library preparation method (Pearson correlation coefficients between 0.912 and 0.996; Fig. 5a). Between the two mRNA library preparation methods, correlations remained high (0.890–0.955; Fig. 5a) with only a small number of genes undetected by one of the mRNA library preparations for each WBC depletion method (Fig. 5b). Overall, gene expression analysis of protein-coding genes yielded similar results, regardless of WBC depletion or mRNA library preparation method.

Across the three library preparation methods, the PMACS method showed the highest reproducibility in the number of genes detected (Fig. 5b). While the PMACS method resulted in a different parasite life stage composition compared to other WBC depletion methods, this did not significantly impact gene expression levels, likely because most parasites in the sample were in the ring or trophozoite stage (correlation coefficients with other WBC depletions with the same library kit range between 0.915 and 0.952, Fig. 5a).

For the total RNA library kit, WBC depletion methods had minimal impact on gene expression levels (correlation coefficients 0.915–0.992, Fig. 5a). However, correlations between total RNA and mRNA library preparations are still significant but much lower (0.569–0.861, Fig. 5a), indicating that total RNA transcriptomes are less comparable to mRNA expression data due to differences in RNA composition, despite a similar number of genes detected between total RNA and mRNA libraries (Fig. 5b).

## Discussion

Our findings highlight the critical importance of WBC depletion and globin RNA depletion for generating high-quality *Plasmodium* transcriptomes and maximizing sequencing output from low-volume natural infections. The impact of WBC depletion methods on the transcriptome profile was minimal, as demonstrated by similarities in the PCA clustering, and the strong correlation in read counts mapped to *P. knowlesi* protein-coding genes. Similarly, the life stage composition did not markedly change after WBC depletion, with the majority of the expression profiles consisting of ring and trophozoite stages. Overall, the consistency in the transcriptome profiles and life stage composition reinforces the reliability of the WBC depletion approaches tested.

When evaluating the different library preparation kits, differences in the transcriptome profiles resulted primarily due to the type of kit used (mRNA vs total RNA), despite the number of detected *P. knowlesi* genes being comparable. The Illumina mRNA kit produced the highest sequencing output (i.e. number of primary mapped PE reads), although these PE reads were heavily dominated by human globin gene counts. In contrast, the Qiagen total RNA kit resulted in the highest number of *P. knowlesi* gene counts; however, these were dominated by *P. knowlesi* rRNA and non-coding RNAs which substantially reduced the proportion of *P. knowlesi* protein-coding gene counts from over 98% to between 8.5 and 13.0% depending on WBC depletion method. While the dominance of rRNA and non-coding RNAs in the Qiagen total RNA kit may be a disadvantage for differential gene expression analysis, it could be useful in investigating non-coding RNAs and processes such as gene regulation.

Among all tested library preparation methods, the Qiagen mRNA kit, including the FastSelect depletion of globin mRNA, yielded the highest number of reads mapping to *P. knowlesi* protein-coding genes, making it the most advantageous for differential gene expression studies. As seen in other studies [44,47, 48], the benefits of globin RNA depletion were evident during library preparation, as it efficiently removed globin RNA and justified its relatively high cost of €48 per sample. Conversely, the addition of an rRNA depletion step in mRNA library preparation did not substantially improve the removal of the relatively small amounts of *Plasmodium* rRNA that remained after poly(A) enrichment. Furthermore, this additional step resulted in some loss of *P. knowlesi*-specific reads without significantly enhancing data quality, rendering the extra cost of €48 per sample unjustifiable. In conclusion, combining globin RNA depletion with an mRNA kit offers the optimal strategy for transcriptomic analyses, particularly for differential gene expression studies, and we recommend it as the preferred approach for future investigations.

Our aim was to increase the feasibility of conducting transcriptomic studies in field settings to lead to a better understanding of *Plasmodium* blood-stage biology. Previous studies proposing transcriptomic protocols for *Plasmodium-*infected blood have focused on different storage conditions and RNA preservation and extraction methods [48,49], leaving WBC depletion methodologies and library preparation kits unexplored. Our study compares different WBC depletion methods suitable for sample collections in the field and evaluates RNA-seq kits with low-volume samples for untargeted *Plasmodium* transcriptomics.

While transcriptomics is relevant to all *Plasmodium* species, studying the transcriptome of blood stages from natural infections is of particular importance for non-*falciparum* species which rely on patient infections to study parasite biology. With *P. vivax* cases reaching 9.2 million cases in 2023 and other non-*falciparum* parasites posing unique challenges to malaria elimination [50], biological insights are crucial to understanding how these parasites are adapting and responding to their changing environments.

*P. vivax* transcriptomic studies from patient isolates have typically relied on *ex vivo* maturation of large volume (>8 ml) venous blood samples [49,51,56]. *Ex vivo* maturation protocols are complex, costly and require specialized expertise and lab facilities that are often unavailable in field settings and likely alter the transcriptome [15,17]. Additionally, when lab facilities are not available near patient recruitment sites, the extra logistics and costs of cryopreservation are also necessary for *ex vivo* maturation [54,56].

In contrast to *ex vivo* maturation, the quick and simple WBC depletion protocols compatible with capillary blood volumes (250 µl) presented here resulted in high-quality comparable expression profiles and are adaptable to collect *Plasmodium* natural infections in the field. To validate the applicability to non-*falciparum* malaria, we used a mock *P. knowlesi* natural infection. *P. knowlesi* is an ideal alternative to *P. vivax* since it closely resembles *P. vivax* morphologically and genetically and has a similar GC nucleotide content in contrast to *P. falciparum*. Currently, our optimized approach combining WBC depletion during sample collection and processing in the field is now being implemented in *P. vivax* transcriptomic field studies in Brazil and Papua New Guinea (data not published).

Only two previous *P. vivax* transcriptomic studies in Cambodia [44,47] have used low-volume (50 µl) capillary whole blood to study natural infections, albeit without WBC depletion. In the study published in 2017, three samples were sequenced using a total RNA kit with globin and rRNA depletion which resulted in 16–30.4% of all PE reads being mapped to *P. vivax* and between 1.6 and 4.0 M PE reads mapping to *P. vivax* protein-coding genes. Our study was able to improve upon this by implementing WBC depletion and an mRNA library preparation kit which increased the protein-coding reads to 5–9 M PE reads (>98% of the parasite reads for each sample), even with similar sequencing output. Additionally, our no filtered samples from the Qiagen total and mRNA kits had a similar percentage of parasite-specific reads (4.5% and 4.7%, respectively) as compared to the study of 26 Cambodia patients which found a median percentage of 4.7% (range 1.1–41.6%) parasite-specific reads [44]. By implementing WBC depletion methods, we were able to obtain a higher percentage of parasite-specific reads in our sequencing output, up to almost 50%. This significant improvement thereby lowers the overall sequencing output and costs required, while closing much of the gap with *P. vivax ex vivo* maturation studies which often report >80% of reads being parasite specific [49,53,55].

When considering the costs associated with WBC depletion, cellulose columns stand out as the most cost-effective option, as they can be assembled in-house using readily available laboratory materials and cellulose powder for less than €0.50 per column. However, this approach requires trained personnel and time to assemble the columns. In contrast, PMACS columns are premade alternatives with user-friendly protocols that can be implemented with minimal prior training. The primary disadvantage is that these options incur significantly higher per-sample costs, €18 and €16 per Plasmodipur filter and MACS column, respectively. NWFs were initially proposed as a less expensive premade alternative to Plasmodipur filters [10]. However, upon inquiry during this study, their actual cost per unit proved to be higher when accounting for shipping. Given their cost, logistical considerations and functional similarity to Plasmodipur filters, NWFs were excluded from our experimental design. For the cost of the library preparation kit, the Qiagen and Illumina library prep kits had the same cost per sample of €57 at the time of purchase for these experiments. If minimizing costs is a priority for the study, cellulose columns are the ideal WBC depletion method, and other options for globin removal kits on the market could be explored.

The discordance between life stage composition determined by microscopy and CIBERSORTx was notable; thus, a more suitable approach would be to use flow cytometry [57], which allows for the quantification of a much larger number of cells, thereby improving the accuracy of the life stage determination. For the PMACS WBC depletion method, which showed an enrichment of the schizont stage by microscopy and CIBERSORTx, we divided the sample in half and passed one half through the MACS column and the other through a Plasmodipur filter. Another approach more representative of the sample composition could have been to run the entire sample on the MACS column first to retain the late stages and subsequently to pass the first elution through the Plasmodipur filter to recover the early stages. Gametocytes are also expected to be captured efficiently by MACS columns [58,60]; however, we could not evaluate their recovery due to the lack of gametocytes in the *P. knowlesi* A1-H.1 strain [24]. While the PMACS method offers advantages for studies focusing on gametocytes or late life stages, its significantly higher cost (€34), time needed and logistical challenges of the strong magnet limit its utility in large-scale transcriptomic studies, particularly in the field.

Additionally, the total RNA kit also appears to contain more schizont life stages in the CIBERSORTx data than the mRNA kits. While unexpected, especially since the deconvolution did not contain rRNA genes, this could be due to the inherent differences found between the two types of kits, as seen in our *P. knowlesi* protein-coding genes PCA, or the signature matrix used for deconvolution. The signature matrix was created from single-cell mRNA transcriptomic data [43]; thus, it is presumably more reflective of the transcriptome profile captured by the mRNA kits. To improve gene expression profile correlations, additional downstream processing tools could be useful to further distinguish coding from non-coding RNA. In our analysis, steps that would normally be enabled in a total RNA analysis pipeline (e.g. annotating lncRNA or miRNA) were disabled to apply the same analysis to all samples. Applying a more traditional total RNA pipeline could thereby result in better comparability between library preparation kits and life stage deconvolution.

In conclusion, we evaluated protocols for processing low-volume *Plasmodium*-infected blood samples for transcriptomic studies, demonstrating their feasibility for both large and small-scale research. The tested WBC depletion methods effectively recovered all parasite life stages present in the samples. The choice of method should be guided by a study’s objectives, laboratory resources, equipment availability and budget, with cellulose columns being the most cost-effective and logistically simple option.

Additionally, we assessed RNA-seq library preparation methods and found that combining mRNA enrichment with globin depletion optimizes sequencing output by removing unwanted RNA and maximizing the read count for *P. knowlesi* protein-coding genes. This approach is particularly recommended for differential gene expression studies. To ensure consistency and minimize variability in sequencing outputs across samples within the same study, it is preferred to use a single RNA-seq library preparation method for all samples.

## Supplementary material

10.1099/mgen.0.001546 Uncited Supplementary Material 1.

## Abbreviations

BH: Benjamini–Hochberg
iRBC: infected red blood cell
NWF: non-woven fabric
PCA: principal component analysis
PE: paired-end
PMACS: Plasmodipur and MACS
RBC: red blood cell
TPM: transcripts per million
WBC: white blood cell
