# Supplementary material for: Low-volume Plasmodium blood sample processing protocols for untargeted transcriptomics optimized using Plasmodium knowlesi
Source: Microb Genom. 2025 Nov 20;11(11):001546. doi: 10.1099/mgen.0.001546 (PMC13293300; doi:10.1099/mgen.0.001546)
Supplement: Uncited Supplementary Material 1. [file mgen-11-01546-s001.pdf]

Supplementary Information

WBC depletion	Library prep kit	FastSelect depletion	RNA Qubit (ng/µL)	V (µL)	Input total ng (Qubit)	Library TS (ng/µL)
no filter	mRNA_illumina	NA	46.9	10	469	8.77
no filter	mRNA_qiagen	globin				4.46
no filter	mRNA_qiagen	globin & human rRNA				1.71
no filter	tRNA_qiagen	globin & human rRNA				10.9
cent/pip	mRNA_illumina	NA	30.3	14	424.2	5.06
cent/pip	mRNA_qiagen	globin				4.49
cent/pip	tRNA_qiagen	globin & human rRNA				9.22
plasmo	mRNA_illumina	NA	17.8	14	249.2	5.92
plasmo	mRNA_qiagen	globin				0.858
plasmo	tRNA_qiagen	globin & human rRNA				7.24
PMACS	mRNA_illumina	NA	10.2	14	142.8	3.96
PMACS	mRNA_qiagen	globin				1.12
PMACS	tRNA_qiagen	globin & human rRNA				5.83
cellulose	mRNA_illumina	NA	11.4	14	159.6	6.02
cellulose	mRNA_qiagen	globin				0.423
cellulose	tRNA_qiagen	globin & human rRNA				2.45

Table S1: RNA extraction and library preparation output concentrations. RNA concentration was measured by Qubit (ng/µL) after RNA extraction and pooling of the three replicates for each WBC depletion method. The volume (µL) added as input and the total nanograms (ng) of RNA used as input to each library preparation kit. The last library concentration is the final concentration after each library preparation and sent for sequencing as quantified by TapeStation (TS) with the D1000 ScreenTape kit.

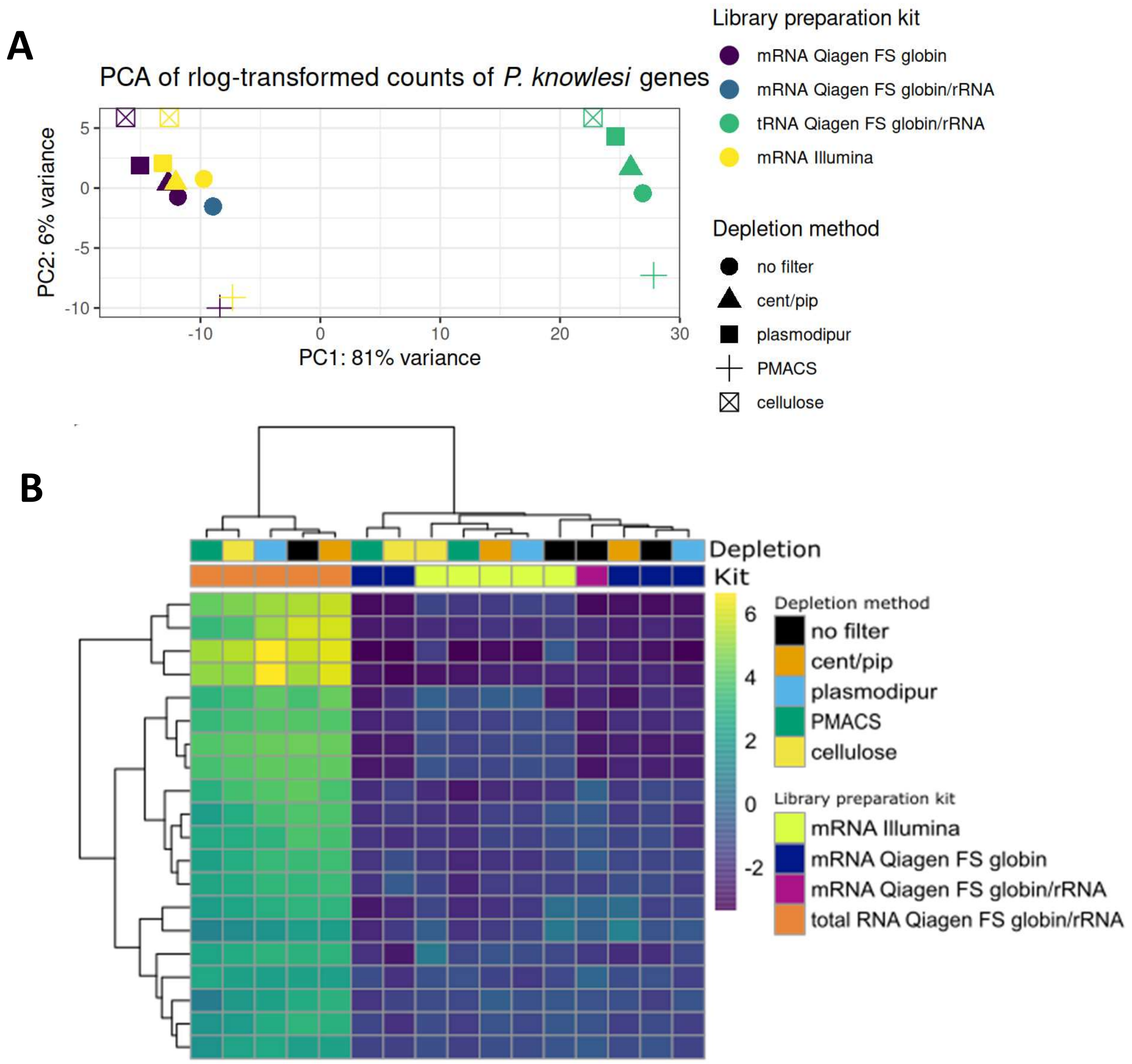

Figure S1: Principal component analysis and heatmap plots of all *P. knowlesi* genes (including rRNA) A) PCA of the top 500 most variable *P. knowlesi* genes including rRNA. B) top 20 most variable *P. knowlesi* genes including rRNA. All gene counts were rlog-transformed after subsetting on the gene list of interest. Low counts were filtered prior to subsetting (minimum count of 10 for at least one sample).

top variable	All *P.knowlesi* gene expression			*P. knowlesi* protein-coding gene expression		
	**gene_id**	**gene_name**	**gene_biotype**	**gene_id**	**gene_name**	**gene_biotype**
1	gene-I6V24_pgp09	gene-I6V24_pgp09	protein_coding	gene-I6V24_pgp09	gene-I6V24_pgp09	protein_coding
2	gene-I6V24_pgp12	gene-I6V24_pgp12	protein_coding	gene-I6V24_pgp12	gene-I6V24_pgp12	protein_coding
3	gene-I6V24_pgr01	gene-I6V24_pgr01	rRNA	gene-I6V24_pgp14	gene-I6V24_pgp14	protein_coding
4	gene-I6V24_pgr02	gene-I6V24_pgr02	rRNA	gene-PKNH_0115400	gene-PKNH_0115400	protein_coding
5	gene-PKNH_0320900	gene-PKNH_0320900	rRNA	gene-PKNH_0401700	gene-PKNH_0401700	protein_coding
6	gene-PKNH_0321000	gene-PKNH_0321000	rRNA	gene-PKNH_0405800	gene-PKNH_0405800	protein_coding
7	gene-PKNH_0321100	gene-PKNH_0321100	rRNA	gene-PKNH_0417000	gene-PKNH_0417000	protein_coding
8	gene-PKNH_0935200	gene-PKNH_0935200	snRNA	gene-PKNH_0914500	gene-PKNH_0914500	protein_coding
9	gene-PKNH_1001000	gene-PKNH_1001000	rRNA	gene-PKNH_0946100	gene-PKNH_0946100	protein_coding
10	gene-PKNH_1001100	gene-PKNH_1001100	rRNA	gene-PKNH_1007800	gene-PKNH_1007800	protein_coding
11	gene-PKNH_1001200	gene-PKNH_1001200	rRNA	gene-PKNH_1010500	gene-PKNH_1010500	protein_coding
12	gene-PKNH_1007800	gene-PKNH_1007800	protein_coding	gene-PKNH_1126200	gene-PKNH_1126200	protein_coding
13	gene-PKNH_1022700	gene-PKNH_1022700	snoRNA	gene-PKNH_1139900	gene-PKNH_1139900	protein_coding
14	gene-PKNH_1271700	gene-PKNH_1271700	ncRNA	gene-PKNH_1409300	gene-PKNH_1409300	protein_coding
15	gene-PKNH_1323850	gene-PKNH_1323850	rRNA	gene-PKNH_1429400	gene-PKNH_1429400	protein_coding
16	gene-PKNH_1339200	gene-PKNH_1339200	ncRNA	gene-PKNH_1451300	gene-PKNH_1451300	protein_coding
17	gene-PKNH_1463550	gene-PKNH_1463550	snoRNA	gene-PKNH_1459000	gene-PKNH_1459000	protein_coding
18	gene-PlknoMp1	cox3	protein_coding	gene-PlknoMp1	cox3	protein_coding
19	gene-PlknoMp2	cox1	protein_coding	gene-PlknoMp2	cox1	protein_coding
20	gene-PlknoMp3	cytb	protein_coding	gene-PlknoMp3	cytb	protein_coding

Table S2: List of the top 20 most highly-variable genes expressed for all *P. knowlesi* genes and *P. knowlesi* protein-coding genes. These lists correspond to the heatmaps of top 20 most highly-variable genes for all *P. knowlesi* genes in Figure S1 and *P. knowlesi protein-coding* genes in Figure 4C and shows that most of the highly-variable genes are primarily rRNA and other non-coding RNAs.
